# An Efficient Lightweight Object Detection Algorithm for Defect Identification in *Paeoniae Radix Alba* With Rapid Area and Diameter Estimation Using OpenCV

**DOI:** 10.1002/fsn3.70947

**Published:** 2025-09-14

**Authors:** Miao Huan, Tao Wang, Qin Xu, Lu Gao, Zhouxiang Lu, Xingyun Shi, Miaohua Qian, Liangquan Jia, Chong Yao

**Affiliations:** ^1^ Huzhou University Huzhou China; ^2^ Huzhou Central Hospital Huzhou China; ^3^ Huzhou Academy of Agricultural Science Huzhou China

**Keywords:** FCA, LSD‐Head, OpenCV, *Paeoniae Radix Alba*, traditional Chinese medicinal, YOLOv8

## Abstract

As an important traditional Chinese medicinal herb, *Paeoniae Radix Alba* (white peony root, WP) not only has significant medicinal value in the field of Chinese medicine but is also widely used in food products and health supplements due to its food and medicine dual‐use properties. The quality of *Paeoniae Radix Alba* is crucial for its efficacy, safety, and effectiveness in food applications, making efficient inspection methods essential. The algorithm uses a self‐developed detection head (LSD‐Head) combined with a Feature Fusion Attention (FCA) mechanism for effective defect detection. Additionally, OpenCV technology is used to accurately measure the physical dimensions of the slices. Compared to the YOLOv8 model (76.4% precision, 63.7% recall, 70.1% mAP@0.5, 8.2 GFLOPs), the proposed model reduces parameter size by 13%, reaching only 65.8% of YOLOv8's size, while improving accuracy by 2.6%. For physical dimensions, the average error between the detected and actual sizes is controlled within 5%. Experimental results show that the proposed algorithm performs well in defect detection and accurately measures the area and maximum and minimum diameters of *Paeoniae Radix Alba* decoction pieces.

## Graphical abstract

EY‐YOLO combines LSD‐Head with Feature Fusion Attention (FCA) to achieve 76.8% precision, 69.7% recall, and 74.1% mAP@0.5, outperforming YOLOv8 (76.4%, 63.7%, 70.1%), while reducing computational cost by 34% (5.4 GFLOPs vs. 8.2 GFLOPs) and parameters by 13% (2.62 M vs. 3.01 M). Combined with OpenCV for physical measurements (< 5% error), this enables efficient dual‐purpose quality control for pharmaceutical and food applications.

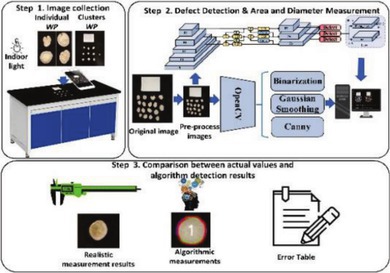

## 1 Introduction

Traditional Chinese medicine (TCM) is essential in promoting and safeguarding public health. With the rising frequency of geriatric diseases and emerging infectious diseases, TCM has garnered increasing attention, and its advanced nature and scientific basis are gaining wider recognition (Chao et al. 2017; Liu and Ma 2020; Hao and Jiang 2015). Chinese medicinal material decoction pieces (CMMDPs) refer to processed herbal products that can be directly used in clinical treatments. Through thousands of years of practice and refinement, CMMDPs have become indispensable in both health preservation and clinical treatment. They are not only an integral part of TCM clinical practice but also play a key role in modern medicine, widely applied to promote health, prevent diseases, and treat various medical conditions (Wong 2021; Rittenhouse and Robinson 2006). Their role in the prevention and treatment of epidemic, chronic, and infectious diseases, such as COVID‐19, has been widely proven and is well recognized (Wang, Liu, et al. 2023).

As demand for high‐quality medicinal herbs grows, attention has increasingly turned toward the efficiency and quality of CMMDPs, with *Paeoniae Radix Alba* being a widely used Chinese medicinal material requiring stringent quality control. Defects in *Paeoniae Radix Alba* decoction pieces refer to conditions such as cracks, insect infestation, hollowing, mold, abnormal color and luster, and mixing of impurities that do not meet quality standards. Factors such as soil fertility, pests and diseases during planting, time of harvesting, slicing and drying techniques of processing, and the environment of storage and transport may lead to the production of these defects. Defects not only affect the appearance of *Paeoniae Radix Alba* decoction pieces and reduce consumer trust, but also destroy the active ingredients, reduce the efficacy of the drug, and even bring potential safety hazards, such as molds that produce toxins and impurities that cause adverse reactions. Meanwhile, the measurement of physical dimensions of *Paeoniae Radix Alba* is of great significance for its quality and grade assessment. Many standards, such as *Bo Paeoniae Radix Alba delivery standard*, *Paeoniae Radix Alba Electronic Trading Specification Standard*, and the group standard *Commodity Specification Grade of Traditional Chinese Medicinal Herbs*—*Paeoniae Radix Alba* (T/CACM 1021.55‐2018), use physical dimensions, such as length and diameter, as the core delineation basis. It proves that the grade quality evaluation strategy established based on physical size measurement is scientific and reasonable.

Traditionally, the defect detection and grading of *Paeoniae Radix Alba* decoction pieces depend on manual, through the naked eye, and experience judgment. This way is not only time‐consuming and inefficient in large‐scale production, but also subject to the subjective factors of the inspectors; different people's judgment standards are different, making it easy to miss and misjudge; it is difficult to ensure the consistency and accuracy of grading. However, with advancements in computer vision and artificial intelligence, automated detection techniques based on image processing and machine learning are opening new possibilities for improving quality control in TCM decoction pieces.

Methods for detecting and recognizing decoction pieces using machine learning are primarily based on extracting key image features, such as color, texture, and shape. These features are then processed using machine learning algorithms for classification and identification (Miao et al. 2023). Techniques commonly applied in the quality assessment of medicinal plants include Partial Least Squares Discriminant Analysis (PLS‐DA) (Cai et al. 2020), Support Vector Machines (SVM) (Venkataraman and Mangayarkarasi 2017), Linear Discriminant Analysis (LDA) (Kaiser et al. 2020), k‐Nearest Neighbors (k‐NN), Random Forest (RF), and Extreme Learning Machines (ELM). While these methods have yielded varying degrees of success across different applications, traditional feature extraction approaches often require intricate engineering and are prone to poor performance under challenging conditions such as complex backgrounds or variable lighting. VanMen et al. (2012) employed several machine learning models, including KNN and SIMCA for classification and PCR and PLS for regression, to analyze LC‐PDA, UV, and FT‐IR data for identifying and quantifying adulterants in Uncaria tomentosa. Their findings showed that UV and LC‐PDA analysis of polyphenols offered strong results in both identification and prediction. Similarly, Yang et al. (2019) trained an SVM model using manually selected NIR and FT‐MIR wavelengths to detect adulterants in Panax notoginseng powder, using data fusion and particle swarm optimization (PSO) to enhance the model's performance. The PSO‐SVM model achieved identification accuracy rates of 96.65% and 96.97%, surpassing the unoptimized models. Kiani et al. (2017) developed a combined system that utilized computer vision and an electronic nose to extract color and aroma features from saffron samples, applying SVM, PCA, and HCA for adulteration detection. Their SVM model achieved high accuracy rates of 89% and 100% in identifying various adulterated saffron samples.

As deep learning technologies continue to advance, deep learning‐based approaches for the detection and recognition of TCM have steadily emerged as a key research focus. These models, built upon multi‐layer neural network architectures, can automatically extract high‐level features from raw data, thereby minimizing the need for manual feature engineering (Ahmed et al. 2023). Numerous traditional deep learning methods have already been applied in medical image analysis, including Convolutional Neural Networks (CNN), Fully Connected Neural Networks (FNN), Recurrent Neural Networks (RNN), Graph Convolutional Networks (GCN), Neural Networks with Random Weights (NNRW), and Deep Convolutional Neural Networks (DCNN). Zhang et al. (2016) utilized Hyperspectral Imaging (HSI) technology combined with intelligent algorithms to distinguish between various parts of *Panax ginseng* , such as the rhizome and main root. By training a YOLO v5s model on a labeled dataset of ginseng images, this method was successfully deployed for object detection, achieving high accuracy, recall, and mean average precision (mAP) of 99.01%, 98.51%, and 99.07%, respectively. This advancement has enabled the intelligent and automated production of *Panax ginseng* . Similarly, Feng et al. (2018). used HSI technology to study damage in winter jujubes from four different sources. Pixel‐level Principal Component Analysis (PCA) was used for qualitative analysis, while SVM, Logistic Regression (LR), and CNN were employed for quantitative analysis. Results indicated that the CNN model performed best, with an accuracy exceeding 85%, and had a short prediction time, providing a theoretical basis for online damage detection of winter jujubes. Wang (2019). improved the feature extraction of CNNs and compared it with other networks such as AlexNet and ResNet‐18. Their approach achieved high classification accuracy and real‐time performance. Huang (2019). modified the LeNet‐5 network to extract relevant features from the leaves of various herbal plants, such as shape and texture differences, which enabled fast recognition with an accuracy exceeding 94%.

With the increasingly urgent need for modernization and development of the traditional Chinese medicine industry, this study focuses on the core objective of establishing a rapid detection system for quality evaluation of *Paeoniae Radix Alba* decoction pieces. A defect detection dataset of *Paeoniae Radix Alba* decoction pieces was constructed to provide a dataset for subsequent research and building on advances in deep learning. This paper proposes an efficient and lightweight defect detection algorithm for *Paeoniae Radix Alba* decoction pieces in response to the demands of edge device deployment and model lightweighting. Combined with OpenCV, it achieves rapid measurement of the leaf area and the maximum to minimum diameters. The proposed algorithm significantly reduces the computational complexity and resource consumption while ensuring the detection accuracy and can adapt to the real‐time detection requirements in the actual production environment.

## 2 Materials and Methods

### 2.1 Materials

#### 2.1.1 Sample Preparation

All samples were provided by Huzhou Central Hospital (Huzhou, China). They were collected from the market and meticulously screened and authenticated by experts to ensure quality and reliability. The samples include *Hangzhou Paeoniae Radix Alba*, *Bozhou Paeoniae Radix Alba*, and *Sichuan Paeoniae Radix Alba*, all of which are medicinal varieties of *Paeoniae Radix Alba*. Defective samples are considered part of these three categories without distinguishing defect types. The identification and verification of these varieties were performed by the pharmacist Yao Jin from *Huzhou Central Hospital*.

The dried samples were taken from whole plant specimens to maintain the stability of their medicinal properties and components. To ensure the freshness and bioactivity of the samples, the dried materials were sealed in vacuum packaging bags and stored under standard refrigerated conditions, with the storage temperature maintained at 6°C (fluctuation range < ±2°C) and relative humidity controlled at 60%–65% (Vera Zambrano et al. 2019). This process was designed to preserve the medicinal value of the samples to the greatest extent, providing a reliable foundation for subsequent research and applications.

#### 2.1.2 Image Data Collection

**FIGURE 1:**
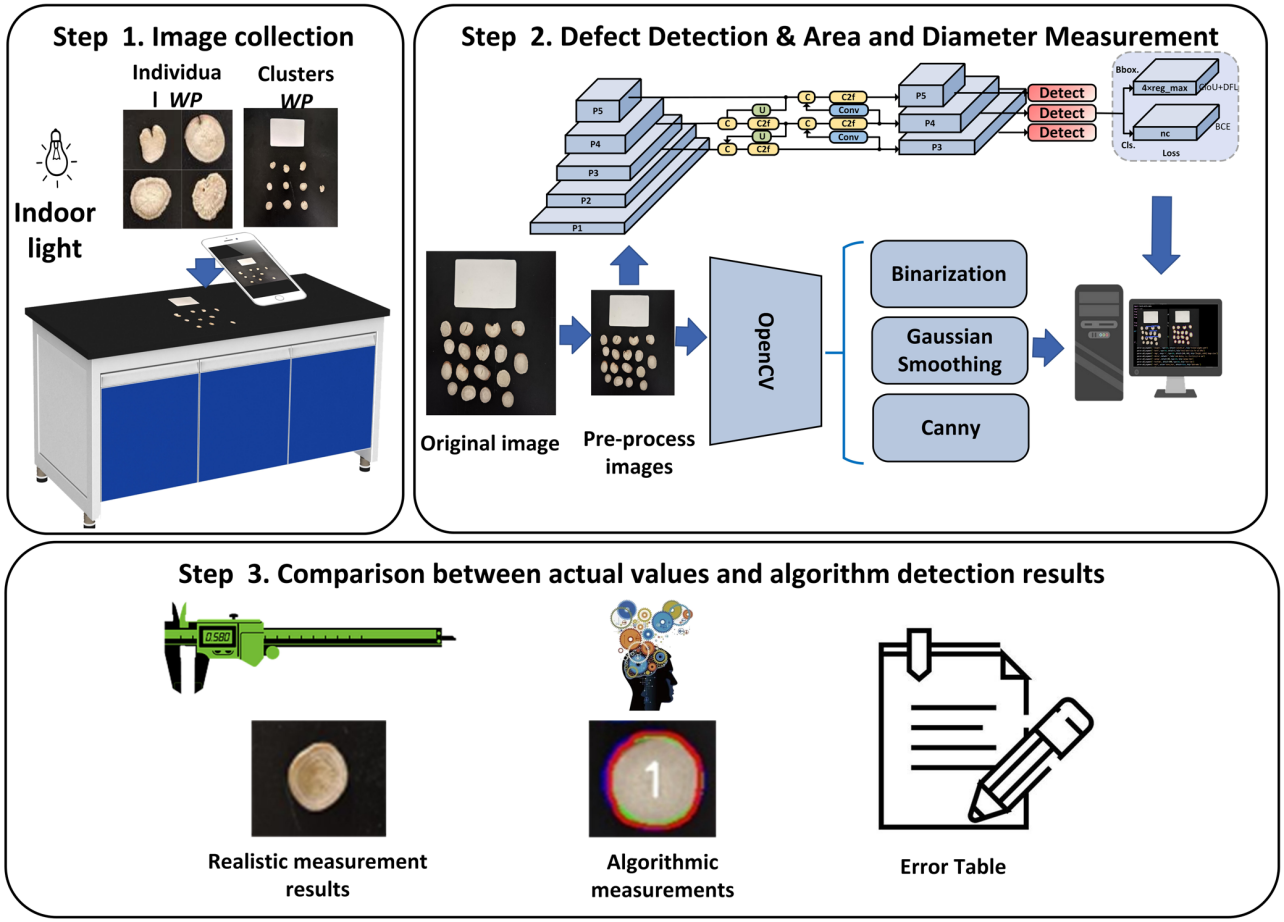
The detection and physical size measurement of *Paeoniae Radix Alba* special‐shaped tablets is the overall process.

High‐quality *Paeoniae Radix Alba* decoction pieces should have a regular slice shape, low crushing rate, uniform thickness of the slices, flat and smooth surface, no cracks, no insect holes, and natural color as the qualifying standards. During the test, white peony slices were placed on the table and photographed with the original camera of a mobile phone. The imaging tool used in this experiment was the Redmi K70 smartphone (main camera: Sony IMX800 sensor, 50 MP resolution, 1/1.49‐in. sensor, f/1.6 aperture, 24 mm equivalent focal length). The shooting settings were: full pixel mode enabled (50 MP output), fixed white balance 5500 K, manual focus mode to lock the shooting distance, exposure compensation set to ±0 EV, and HDR function disabled. The shooting environment was an indoor incandescent light source (Color temperature 2700 k, lumen 500 lux); the background was backed by two types of backplates, pure black and green respectively, and the shooting distance was fixed at 30 cm, and each image contained varying numbers of *Paeoniae Radix Alba* decoction pieces. A piece of cardboard, the same size as a Chinese national ID card (26 × 38 mm), was placed above the *Paeoniae Radix Alba* decoction pieces. The images were taken at the same light source, at different times, from different angles, and with different backgrounds. A total of three categories of *Paeoniae Radix Alba* were captured, and after manual screening, 1200 high‐quality images were selected for the experiment. Each variety contained 400 images, with an average of 200 images per variety on each of the two backgrounds. The original resolution of the collected images was 3072 × 3072 pixels. For the convenience of model training, the *Paeoniae Radix Alba* images were resized to 640 × 640 pixels. An example of the finalized images used for model training is shown in Step 1 of Figure 1 Image Collection.

### 2.2 Overall Network Structure

R‐CNN and YOLO are two primary deep learning approaches for object detection, both utilizing the idea of candidate regions to generate potential bounding boxes. They generate potential candidate boxes or grids that may contain target objects using different methods (Maity et al. 2021). The R‐CNN family of algorithms employs a two‐stage detection process: initially, candidate regions are generated, which are then classified and refined through bounding box regression. Although this method excels in localization accuracy, it suffers from low computational efficiency due to the need to process a large number of candidate regions individually. In contrast, the YOLO series of algorithms employs a single‐stage detection strategy, directly generating object detection results on the image, thereby significantly reducing the computational burden and improving detection speed. Its strong real‐time performance makes it widely used in real‐time image recognition.

YOLOv8 (Wang, Chen, et al. 2023) inherits the core concepts of YOLOv3 (Zhao and Li 2020) and YOLOv5, while making significant improvements. It features state‐of‐the‐art (SOTA) models, such as the P5 640 and P6 1280 object detection networks, along with an instance segmentation model based on YOLACT (Bolya et al. 2019). Similar to YOLOv5, YOLOv8 provides models in various sizes (N/S/M/L/X) to accommodate different scene requirements. Furthermore, YOLOv8 replaces the C3 structure from YOLOv5 with the C2f structure for enhanced gradient flow and boosts performance by fine‐tuning across different model scales. In the detection head, YOLOv8 adopts a separate classification and detection head structure, transitioning from an anchor‐based approach to an anchor‐free design. Finally, YOLOv8 introduces the Task‐Aligned Assigner strategy for positive sample assignment and employs Distribution Focal Loss to compute losses, resulting in significant improvements in detection performance.

**FIGURE 2:**
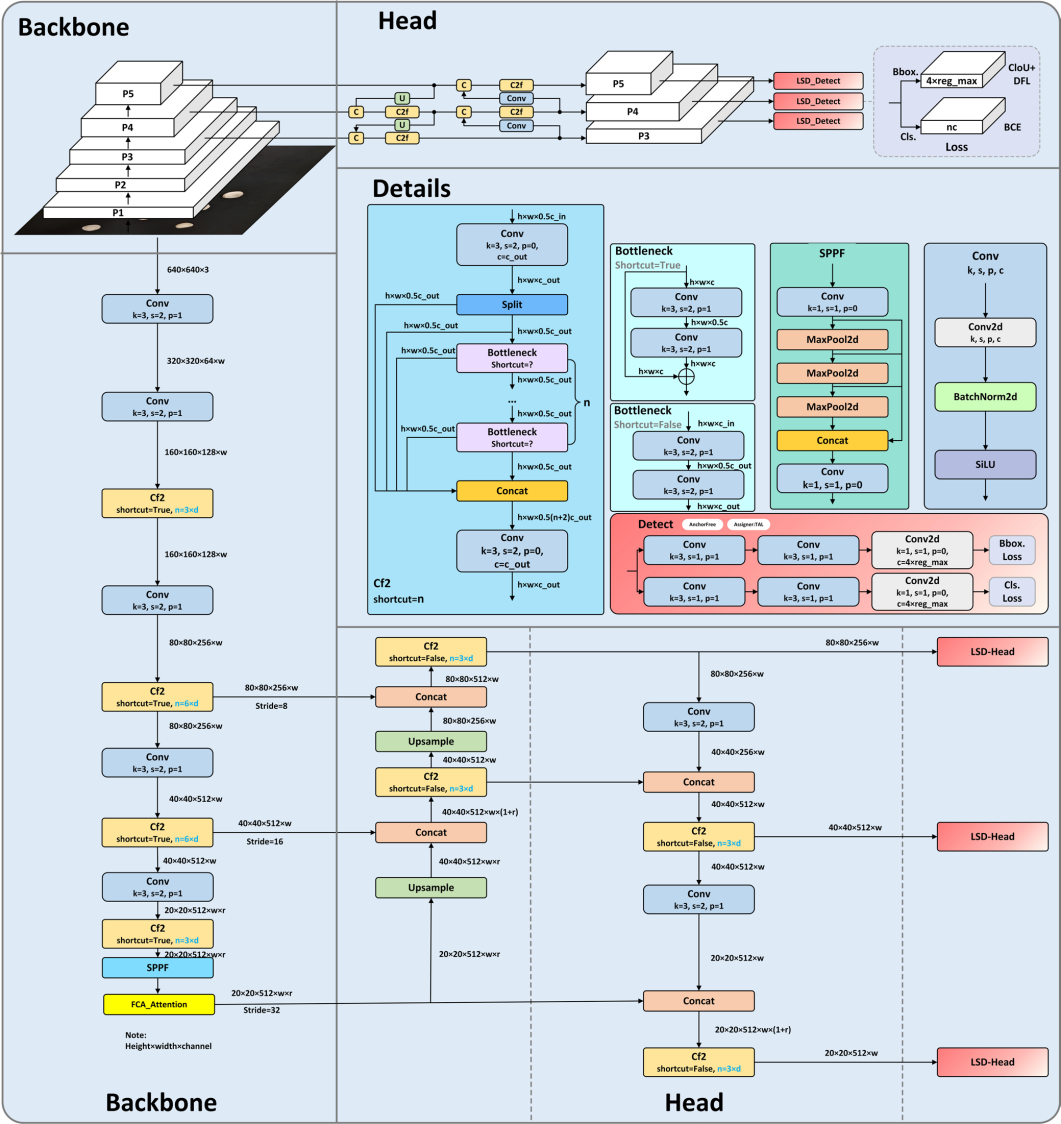
Overall model structure diagram for defect detection in *Paeoniae Radix Alba*.

To achieve real‐time defect detection for *Paeoniae Radix Alba* and ensure practical deployability while minimizing computation and deployment costs, this paper selects the lightest model in the YOLOv8 series, YOLOv8n, as the base model. Due to its lightweight characteristics, YOLOv8n is optimized for mobile devices with constrained processing power and memory. However, to further improve the performance of the defect detection model for *Paeoniae Radix Alba*, we propose a new detection model based on YOLOv8n. The new model swaps out YOLOv8's original detection head with a self‐developed lightweight detection head featuring shared parameters. Additionally, the latest FCA (Sun et al. 2024) module is introduced into the high‐level semantic information module to enhance the model's ability to capture high‐level semantic information. Compared to the original model, the proposed model demonstrates a lower parameter count and computational cost in the *Paeoniae Radix Alba* defect detection task, while significantly improving detection accuracy, showcasing its potential advantages in practical applications. The overall structure of the model is shown in Figure 2.

#### 2.2.1 Self‐Developed Lightweight Detection Head

In the YOLOv8 model, the Head has undergone significant changes, transitioning from a coupled head design to a decoupled one, and shifting from the anchor‐based detection method of YOLOv5 to an anchor‐free approach. The new decoupled head eliminates the traditional objectness branch and directly separates into a regression branch and a classification branch. The regression branch uses an integral representation technique introduced by Distribution Focal Loss. These improvements have significantly enhanced the performance of YOLOv8. However, to meet the specific needs of *Paeoniae Radix Alba* detection, this study proposes the LSD‐Head structure, a lightweight shared detail‐enhanced convolutional detection module. Inspired by models such as RTMDet (Lyu et al. 2022), the LSD‐Head is designed to balance detection accuracy and computational efficiency through structural simplification and enhanced feature sharing, making it particularly suitable for resource‐constrained environments. By employing 1 × 1 convolution layers to effectively reduce the number of channels in the input feature maps, LSD‐Head significantly reduces computational overhead. Its core component—the shared detail‐enhanced convolutional layer (DEConv_GN)—improves feature consistency and reduces parameter redundancy by sharing rich feature representations. This allows the model to maintain high detection accuracy while substantially lowering computational complexity. Additionally, the baseline model uses convolutional steps with pooling downsampling, which can lead to the loss of details, spatial information, and degradation of feature expressiveness. However, the LSD‐Head detector head designed in this paper can effectively enhance the image information by adopting multi‐dimensional feature fusion.

The LSD‐Head structure also includes separate regression and classification layers. The regression layer uses the reg_max strategy to generate precise bounding box predictions, while the classification layer outputs the confidence score for each target variety. To adapt to inputs of different resolutions, LSD‐Head introduces a scale adjustment mechanism that dynamically adjusts the scale of feature maps, ensuring consistency in multi‐scale detection tasks. Additionally, the LSD‐Head leverages advanced techniques from RTMDet in label assignment and training strategies, incorporating a dynamic soft label assignment strategy to stabilize label matching during training, which improves the model's convergence speed and final accuracy.

These enhancements result in significant improvements in both efficiency and accuracy, and LSD‐Head exhibits strong scalability, making it applicable to various object detection tasks. By integrating state‐of‐the‐art object detection technologies, particularly key design concepts from RTMDet, LSD‐Head provides a high‐efficiency, high‐accuracy solution for modern object detection tasks, making the *Paeoniae Radix Alba* detection model more feasible for use in real‐world production environments.

(1) $$F_{\text{out}}=\text{DEConv}\left(F_{\text{in}}\right)=\sum_{i=1}^{5}F_{\text{in}}\times K_{i}=F_{\text{in}}\times \left(\sum_{i=1}^{5}K_{i}\right)=F_{\text{in}}\times K_{\mathrm{cvt}}$$

**FIGURE 3:**
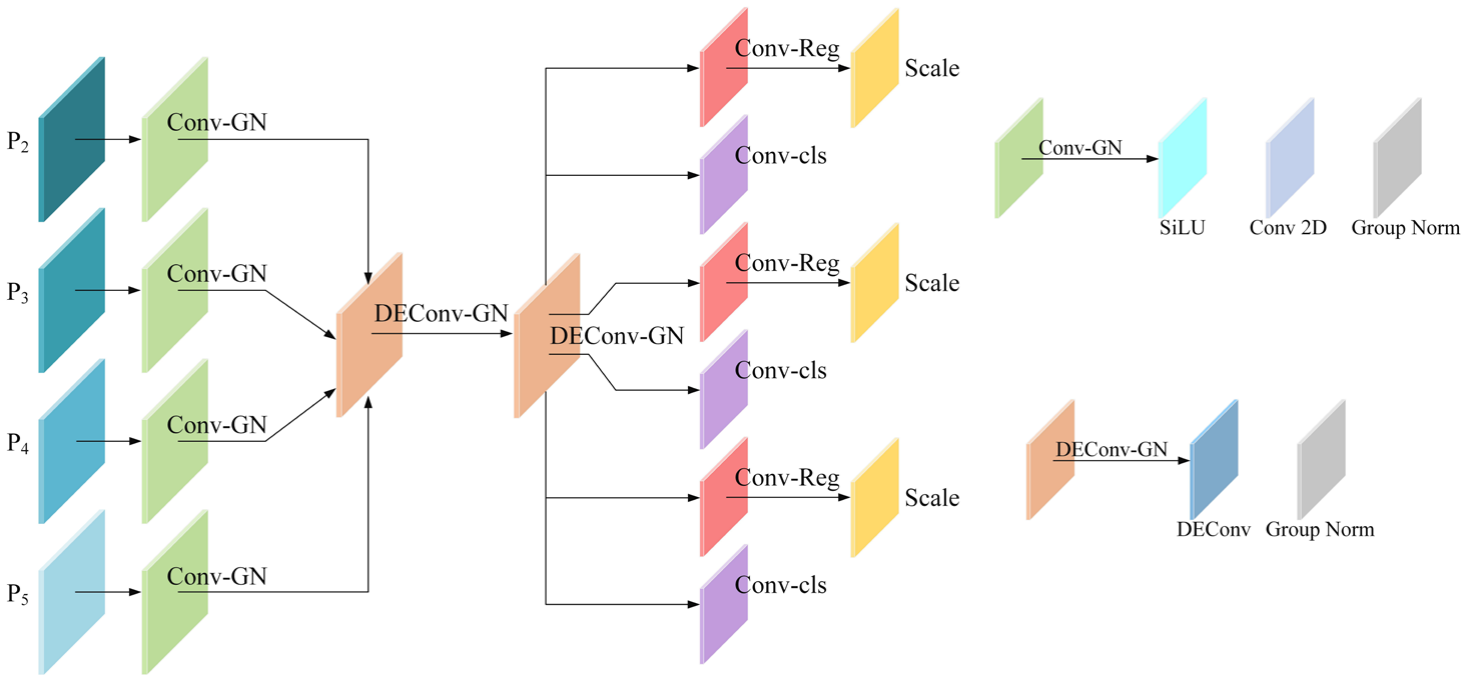
LSD‐Head block.

In Figure 3, the key component of the LSD‐head is the DEConv_GN module, where the DEConv module enhances the flexibility of convolution operations by integrating multiple types of convolutions, thereby optimizing feature extraction. The DEConv module includes five custom convolution layers: Center Difference Convolution, Horizontal Difference Convolution, Vertical Difference Convolution, Adaptive Difference Convolution, and the standard 2D convolution layer. Since five parallel convolutional layers increase the number of parameters and inference time, using the additivity of the convolutional layers, the kernel weights of the five parallel convolutions are updated according to the chain rule of gradient propagation in the back‐propagation stage; while in the forward propagation stage, the kernel weights of the parallel convolutions are fixed, and the equivalent kernel weight of the merged convolutional layers are computed, which achieves the extraction of richer features without increasing the number of parameters and the cost of computation The model can be used to extract more features without increasing the number of parameters and computational cost, while accelerating the training and testing process, allowing the model to have stronger feature extraction capability while maintaining high efficiency. The above process is shown in Equation (1), where $K_{i}$ represents the convolution kernels of different convolutional layers, * is the convolution operation, and $K_{\mathrm{cvt}}$ is the equivalent convolution kernel merged after reparameterisation. The advantage of this design lies in its ability to capture feature information from multiple perspectives, and during inference, these convolution operations are unified into a single convolution operation, improving the model's inference efficiency. As can be seen from Table 1, the mAP@0.5 (%) is 70.1 when using YOLOv8 only, and improves to 72.7 after the introduction of LSD—Head, which reflects its comprehensive feature capturing capability effectively improves the detection accuracy. Meanwhile, multiple convolutions are fused into one during inference, which reduces the computation amount. The GFLOPs are 8.2 in YOLOv8 only, but reduced to 5.4 after adding LSD‐Head, and the number of parameters is reduced from 3.01 to 2.56 M, which significantly improves the computation efficiency.

**TABLE 1:** Ablation study between different modules.

YOLOv8	LSD‐Head	FCA	Precision (%)	Recall (%)	mAP@0.5 (%)	GFLOPs	Parameter (M)
✓			76.4	63.7	70.1	8.2	3.01
✓	✓		77.9	64.3	72.7	5.4	2.56
✓	✓	✓	76.8	69.7	74.1	5.4	2.62

#### 2.2.2 Adaptive Fine‐Grained Channel Attention Module

High‐level semantic information is crucial for distinguishing between different categories of objects. In high‐level features, the model converts low‐level details (such as edges and textures) into more discriminative semantic features through multiple convolution and pooling operations, enabling more accurate object classification. From YOLOv5 to YOLOv8, the SPPF (Spatial Pyramid Pooling Fast) module has been added after the high‐level semantic information extraction module. SPPF is a fast version of spatial pyramid pooling, a technique used to handle inputs of different sizes. The core idea of SPPF is to generate fixed‐length feature vectors by performing pooling operations at different scales on the input feature map, allowing the network to process images of arbitrary sizes. However, after multiple layers of pooling and convolution, some of the original semantic information may be lost. To address this issue, this paper introduces an Adaptive Fine‐Grained Channel Attention (FCA) mechanism after the SPPF structure to enhance the fusion capability of high‐level semantic information.

**FIGURE 4:**
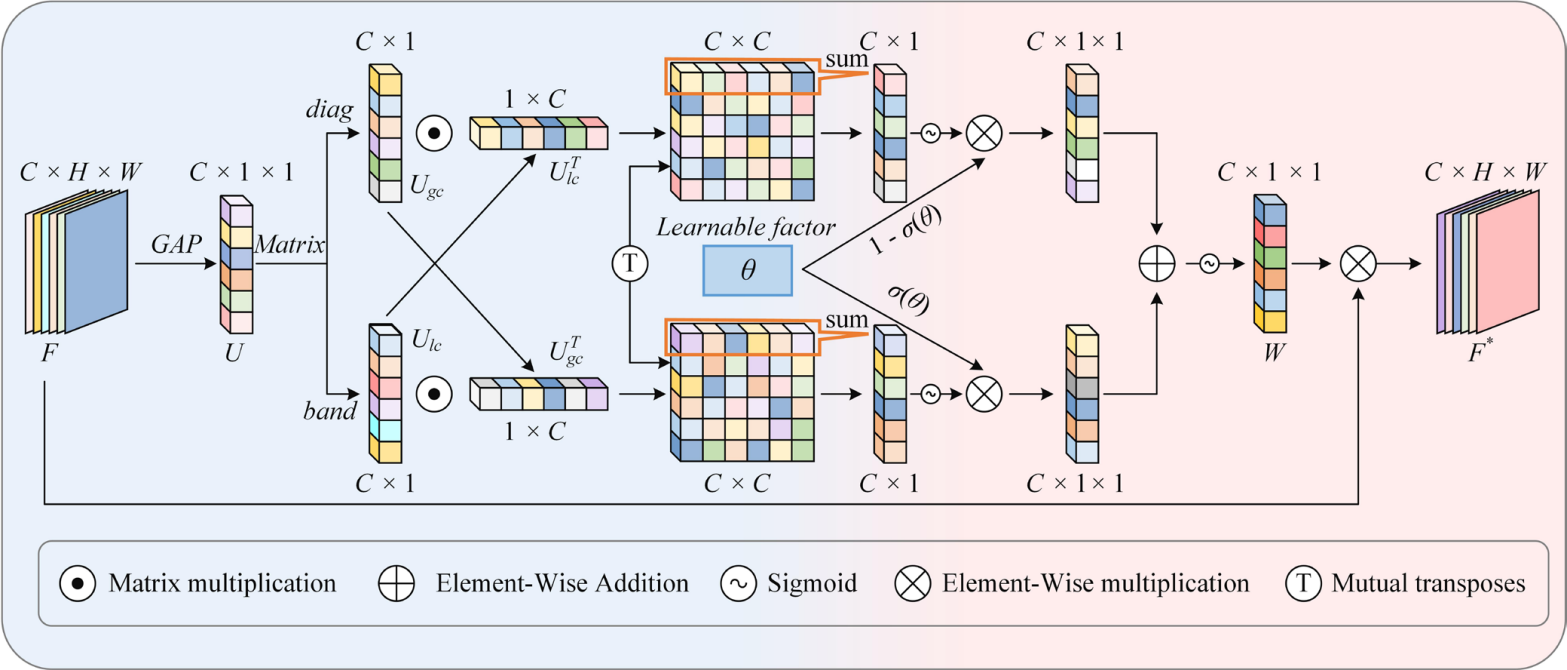
FCA mechanism.

The FCA mechanism is a novel attention module proposed by Sun et al. (2024). in the UBRFC‐Net network. It aims to improve network performance by dynamically adjusting the channel weights of feature maps by combining global and local information. First, FCA uses global average pooling to extract global information from each channel and captures local correlations between channels through a 1D convolution. Next, a cross‐correlation operation is used to combine global and local information, generating a correlation matrix to capture dependencies at different granular levels. FCA adopts an adaptive fusion strategy, dynamically adjusting the weight ratio of global and local information through learned factors and applying the fused weights to the input feature map, enabling more precise channel feature extraction. Figure 4 illustrates the FCA mechanism structure, and Equations ((2), (3), (4), (5), (6), (7)) explain the mathematical principles of FCA.

(2) $$\begin{array}{c} U_{n}=\mathrm{GAP}\left(F_{n}\right)=\frac{1}{H\times W}\sum_{i=1}^{H}\sum_{j=1}^{W}F_{n}\left(i,j\right) \end{array}$$

(3) $$\begin{array}{c} U_{\mathrm{lc}}=\sum_{i=1}^{k}U\cdot b_{i} \end{array}$$

(4) $$\begin{array}{c} U_{\mathrm{gc}}=\sum_{i=1}^{c}U\cdot d_{i} \end{array}$$

(5) $$\begin{array}{c} M=U_{\mathrm{gc}}\cdot U_{\mathrm{lc}}^{T} \end{array}$$

(6) $$\begin{array}{c} W=\sigma \left(\sigma \left(\theta \right)\times \sigma \left(U_{w}^{\mathrm{gc}}\right)+\left(1-\sigma \left(\theta \right)\right)\times \sigma \left(U_{w}^{\mathrm{lc}}\right)\right) \end{array}$$

(7) $$\begin{array}{c} F^{*}=W⊗F \end{array}$$

In the FCA (Feature Fusion Attention) mechanism, Equation (2) extracts global information from each channel via global average pooling, reducing the spatial dimensions of the input feature map to 1 × 1. Equation (3) employs 1D convolution to extract local channel information, capturing the local correlation between adjacent channels. Equation (4) further extracts global channel information through a diagonal matrix. Equation (5) combines global and local information via a cross‐correlation operation, generating a correlation matrix that captures information dependencies at different granularities. Equation (6) dynamically fuses global and local information through the sigmoid activation function and a learnable factor, *θ*, calculating the final channel weight, W. Finally, Equation (7) applies these weights to the input feature map, producing the weighted output feature map $F^{*}$, thereby achieving precise feature extraction and enhancement.

### 2.3 Edge Detection Using OpenCV

This algorithm leverages computer vision techniques for precise measurement of objects in images, making it particularly suitable for measuring the area, minimum diameter, and maximum diameter of *Paeoniae Radix Alba* decoction pieces. First, the algorithm converts the input color image into a grayscale image to simplify subsequent processing and uses a Gaussian blur filter to reduce noise interference, thereby improving the accuracy of edge detection. Next, the Canny edge detection algorithm is employed to extract significant edges from the image, identifying the contours of objects. By setting dual thresholds, the algorithm effectively suppresses insignificant edges while enhancing the visibility of key edges.

Among the detected contours, the algorithm filters out potential reference objects, such as an identification card, by calculating the aspect ratio of the bounding box. Since the actual dimensions of the identification card are known, the algorithm can accurately identify its contour and calculate the pixel‐to‐millimeter ratio in the image by matching its aspect ratio. This ratio serves as the basis for subsequent measurements, allowing the algorithm to convert pixel units in the image into actual physical dimensions.

After identifying the reference object, the algorithm iterates through other contours in the image, calculates the width and height of their bounding boxes, and converts these measurements into millimeter units, thereby determining the actual size of each object. The algorithm finally displays these measurement results on the image, including the area, minimum diameter, and maximum diameter, providing an accurate method for object measurement and analysis. This approach is well suited for automated processing of large‐scale image data, offering high repeatability and reliability.

In this experiment, the geometric properties of *Paeoniae Radix Alba* were measured using OpenCV, achieving accurate calculations of its area, minimum diameter, and maximum diameter through computer vision techniques.

(8) $$\begin{array}{c} \left(x,y\right)=\left\{\begin{array}{cc} 1 & \text{if}\nabla I\left(x,y\right)\geq T\\ 0 & \text{if}\nabla I\left(x,y\right)<T \end{array}\right. \end{array}$$

(9) $$\begin{array}{c} \mathrm{PPM}=\frac{W_{\text{pixels}}}{W_{\mathrm{mm}}} \end{array}$$

Equation (8) represents the edge detection formula, where “$\nabla I\left(x,y\right)$” denotes the magnitude of the image gradient, and “T” is the threshold value set for edge detection. Equation (9) refers to the PPM (Pixels Per Meter) ratio, which is the ratio between the image width in pixels and the actual physical dimensions. In this equation, “$W_{\text{pixels}}$” represents the width of the ID card in the image in pixels, and “$W_{\mathrm{mm}}$” represents the actual width of the ID card.

### 2.4 Model Evaluation Metrics

In this experiment, we performed a comprehensive evaluation of the defect detection model for substandard *Paeoniae Radix Alba (white peony)* products, using various evaluation metrics, including precision, recall, mean average precision (mAP), the number of parameters, and floating‐point operations per second (GFLOPs).

(10) $$\begin{array}{c} \text{Precision}=\frac{\mathrm{TP}}{\mathrm{TP}+\mathrm{FP}} \end{array}$$

Recall measures the proportion of true positive samples among all actual positive samples, indicating the model's ability to comprehensively detect defects. The formula is as follows:

(11) $$\begin{array}{c} \text{recall}=\frac{\mathrm{TP}}{\mathrm{TP}+\mathrm{FN}} \end{array}$$

In this experiment, mAP (Mean Average Precision) is calculated by averaging the precision of all defect categories to evaluate the model's performance in multi‐class defect detection tasks:

(12) $$\begin{array}{c} \mathrm{mAP}=\frac{1}{n}\sum_{i=1}^{n}\;{\mathrm{AP}}_{i} \end{array}$$

In this context, TP (True Positives) refers to the number of defect areas in the *Paeoniae Radix Alba* that the model correctly detected, representing the defects that the model accurately identified. On the other hand, FP (False Positives) refers to the number of times the model incorrectly identified non‐defective areas as defective. FN (False Negatives) represents the number of defect areas that the model failed to detect, meaning the actual defects that the model missed.

## 3 Results and Discussion

### 3.1 Experimental Platform and Parameter Settings

In this experiment, the model training was conducted on a Linux operating system using the PyTorch framework for both training and evaluation. The server was equipped with an Intel(R) Xeon(R) CPU E5‐2650 v3 @ 2.30GHz processor, 64GB of RAM, and an NVIDIA V100 GPU with 32GB of memory to accelerate computations. The system was configured with the CUDA 11.2 parallel computing framework and CUDNN 8.2 deep learning library to ensure efficiency and robustness throughout the training process. The input image resolution was set to 640 × 640 pixels, with a batch size of 32, and the total number of training steps was 200. The learning rate was set to 0.01, with a momentum value of 0.937. Stochastic gradient descent (SGD) was chosen as the optimizer, and the weight decay was set to 0.005.

For local testing, validation was performed using a Windows 11 operating system, with hardware including an Intel Core i3 12100 processor, 16GB of RAM, and a GeForce GTX 3060 GPU with 12GB of VRAM. Similarly, the local environment was configured with the CUDA 11.2 framework and the CUDNN 8.2 library to accelerate inference and validation tasks.

### 3.2 Ablation Experiments

To verify the effectiveness of the self‐developed lightweight detection head, LSD‐Head, and the FCA mechanism in *Paeoniae Radix Alba* defect detection, as well as the overall effectiveness of the proposed *Paeoniae Radix Alba* detection model, a series of ablation experiments were conducted. These experiments included ablation tests between different modules, ablation tests for different attention mechanisms, and ablation tests for various loss functions. The experimental results are shown in Tables 1, 2, 3.

Based on the analysis of Table 1, it can be observed that after improving the detection head of the original model with the LSD‐Head module, the model's parameter count was reduced by approximately 15%. Despite the lightweight modifications, the model achieved unexpected results: the mAP value not only did not decrease but increased by 2.6 percentage points. On this basis, the research team introduced the latest attention mechanism, which integrates both global and local information. Ultimately, our *Paeoniae Radix Alba* detection model achieved a 4‐percentage‐point improvement in accuracy compared to the original model, with computational cost reduced to only 65.8% of the original, and the model's parameter count reduced by 13%.

To validate the superiority of the FCA attention mechanism used in our proposed *Paeoniae Radix Alba* defect detection scheme, we conducted a comparative study with several attention mechanisms that have attracted significant attention in recent years, including SimAM (Yang et al. 2021), TA (Shi 2024), Multilinear Principal Component Analysis (MPCA) (Chen et al. 2023), Exponential Moving Average (EMA) (Ouyang et al. 2023), and CA (Hou et al. 2021). According to the experimental results in Table 2, the FCA attention mechanism achieved an accuracy of 74.1% in *Paeoniae Radix Alba* defect detection, significantly outperforming other methods. These results strongly demonstrate the exceptional performance of the FCA attention mechanism in *Paeoniae Radix Alba* defect detection, making it the optimal choice at present.

**TABLE 2:** Ablation study of different attention mechanisms.

YOLOv8‐LSD	SimAM	SE	MPCA	TA	CBAM	EMA	CA	FCA	mAP@0.5 (%)	Racall (%)
✓									72.7	64.3
✓	✓								71.7	67
✓		✓							71.9	63.8
✓			✓						72.1	63.6
✓				✓					72.2	65.3
✓					✓				72.4	65.1
✓						✓			73.5	66.2
✓							✓		73.7	68.8
✓								✓	74.1	69.7

Based on the analysis of Table 3, in the IoU selection part of the model, this experiment compared the currently common IoU metrics. The results showed that the original CIoU loss function is highly sensitive to *Paeoniae Radix Alba* defect detection. Using the CIoU loss function, the *Paeoniae Radix Alba* model achieved a mAP@0.5 of 74.1% and a recall rate of 69.7, offering the highest precision and recall rates. This makes the CIoU loss function the optimal choice for defect detection in *Paeoniae Radix Alba* at present.

**TABLE 3:** Ablation study among different IoU modules.

IoU loss function	mAP@0.5 (%)	Recall (%)
CIoU	74.1	69.7
EIoU	72.1	65.3
DIoU	72.5	67.1
SIoU	72.8	66.9
GIoU	72.5	68.1

### 3.3 Comparative Experiments

To verify the superiority of the proposed defect detection model for *Paeoniae Radix Alba*, we conducted systematic and comprehensive experimental comparisons on a self‐constructed *Paeoniae Radix Alba* dataset. Specifically, we selected several state‐of‐the‐art detection models for comparative analysis, including classic convolutional neural network models such as YOLOv5, YOLOv7, and YOLOv10, as well as the Transformer‐based detection model RT‐DETR. The experimental results, as shown in Table 4, clearly demonstrate the outstanding performance of the proposed model in the *Paeoniae Radix Alba* defect detection task, further proving its significant advantage in this field.

**TABLE 4:** Comparative experiments of different models.

Module	mAP@0.5 (%)	GFLOPs	Parameter (M)
YOLOv8	70.1	8.2	3.01
YOLOv5	69.8	7.2	2.5
YOLOv10	68.1	8.4	2.7
RT‐DETR	73.8	58.6	20.8
YOLOv8s	72.3	28.6	11.4
YOLOv8m	74.4	78.9	26.2
YOLOv8l	75.2	165.4	43.7
EF‐Yolo	74.1	5.4	2.62

**FIGURE 5:**
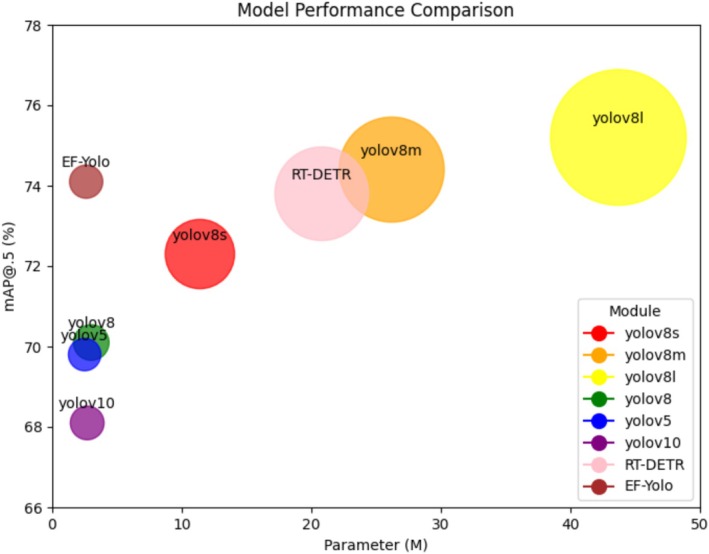
Comparison of parameters and performance of different models.

Based on the analysis results from Table 4 and Figure 5, our proposed EF‐YOLO model demonstrates outstanding overall performance in the task of *Paeoniae Radix Alba* defect detection, showing significant advantages in terms of accuracy, parameter count, and computational cost. Compared to the state‐of‐the‐art YOLOv10 model, the accuracy of the EF‐YOLO model is improved by 6 percentage points. Additionally, compared to the Transformer‐based RT‐DETR model, the parameter count and computational cost of EF‐YOLO are significantly reduced, indicating that the model can achieve efficient *Paeoniae Radix Alba* defect detection with lower computational resources while maintaining high accuracy.

### 3.4 Experimental Analysis of *Paeoniae Radix Alba* Defect Detection Model

#### 3.4.1 Experiment Visualization Results Output

**FIGURE 6:**
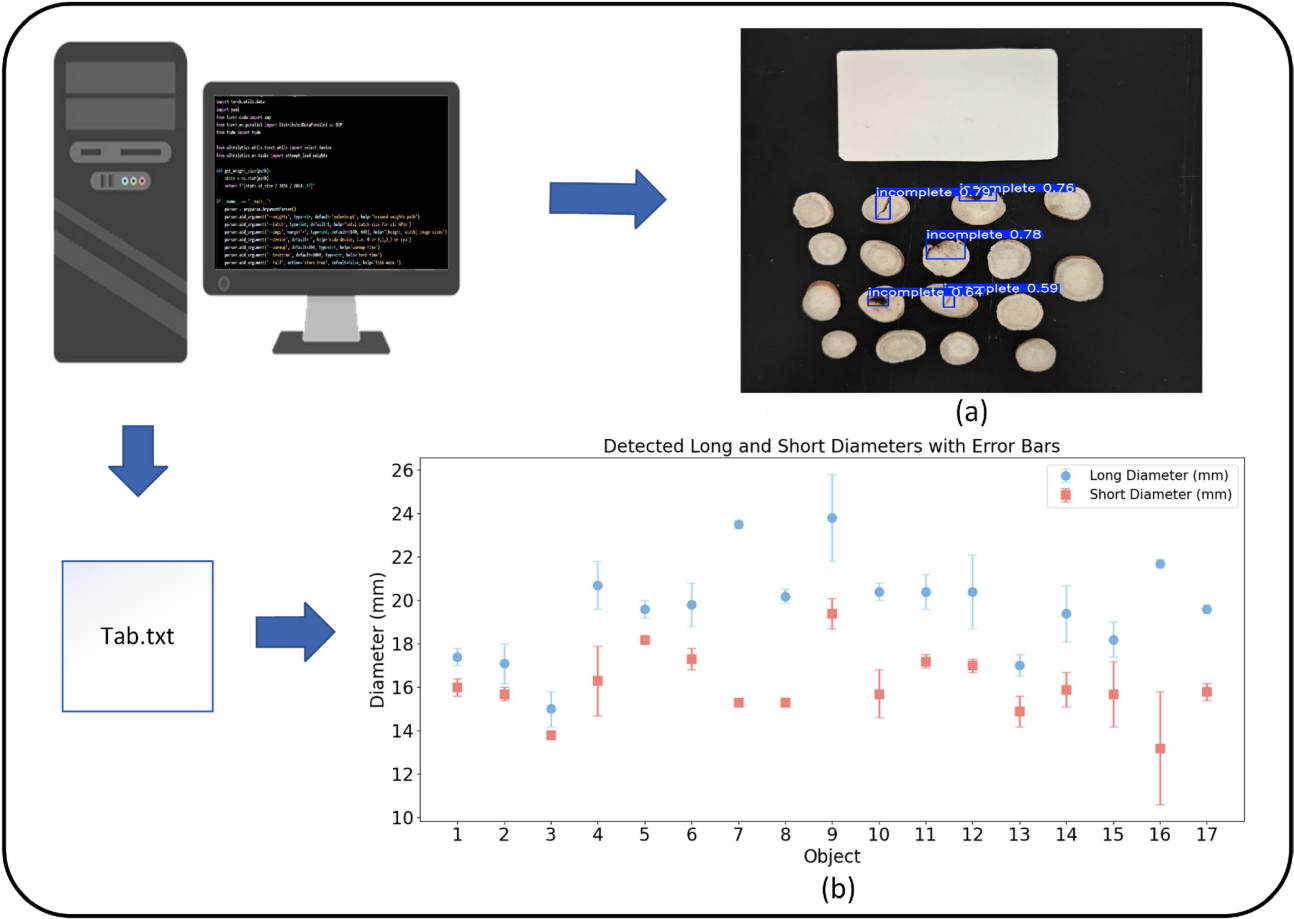
(a) Defect detection result image; (b) error plot between true and detected values.

After training the *Paeoniae Radix Alba* detection model, the research team integrated the OpenCV‐based physical attribute detection method with the model's prediction process. In practical applications, only the trained detection model needs to be loaded to automatically generate two result images: one for defect detection and the other for the physical dimension identification of *Paeoniae Radix Alba*. Finally, the model also generates a text file (txt) that corresponds to the *Paeoniae Radix Alba* physical dimension identification image, recording the relevant detection data. Figure 6a shows the defect detection result, while Figure 6b displays the *Paeoniae Radix Alba* identification for physical properties. Table 5 lists the key parameters of *Paeoniae Radix Alba*, including the shortest diameter, longest diameter, and area. Among them, Long‐Error and Short‐Error represent the error rate between the actual physical dimensions and the predicted values, while Long‐Acc and Short‐Acc indicate the accuracy of the detection results.

**TABLE 5:** Comparison and error analysis table of actual physical dimensions of *Paeoniae Radix Alba* and OpenCV detection results.

Object	Long‐diameter (mm) (actual)	Short‐diameter (mm) (actual)	Long‐diameter (mm) (prediction)	Short‐diameter (mm) (prediction)	Area (mm^2^) (prediction)	Long‐error (%)	Short‐error (%)	Long‐Acc (%)	Short‐Acc (%)
1	17.0	15.6	17.4	16.0	215.3	2.4	2.6	97.6	97.4
2	16.2	15.4	17.1	15.7	204.8	5.6	1.9	94.4	98.1
3	14.2	13.6	15	13.8	157.8	5.6	1.5	94.4	98.5
4	19.6	14.7	20.7	16.3	266.8	5.6	10.9	94.3	89.1
5	19.2	18.3	19.6	18.2	275.7	2.1	0.5	97.9	99.5
6	18.8	16.8	19.8	17.3	262.3	5.3	3.0	94.7	97.0
7	23.3	15.2	23.5	15.3	284.6	0.9	0.7	99.1	99.3
8	19.9	15.1	20.2	15.3	239.7	1.5	1.3	98.5	98.7
9	21.8	18.7	23.8	19.4	362.7	9.2	3.7	90.8	96.3
10	20.0	14.6	20.4	15.7	242.9	2.0	7.5	98.0	92.5
11	19.6	16.9	20.4	17.2	274.2	4.1	1.8	95.9	98.2
12	18.7	17.3	20.4	17.0	272.3	9.1	1.7	90.9	98.3
13	16.5	14.2	17	14.9	195.2	3.0	4.9	97.0	95.1
14	18.1	15.1	19.4	15.9	239.1	7.2	5.3	92.8	94.7
15	17.4	14.2	18.2	15.7	221.8	4.6	10.6	95.4	89.4
16	21.5	15.8	21.7	13.2	265.5	0.9	16.5	99.0	83.5
17	19.4	15.4	19.6	15.8	244.6	1.0	2.6	99.0	97.4

#### 3.4.2 Analysis of Defect Detection and OpenCV Results

As can be seen from the Figure 6a, the detection of *Paeoniae Radix Alba* samples by the established system is able to identify whether there are defects in the samples, which are clearly labeled in the resultant images. It shows that the defect detection algorithm has the ability to identify the defects in *Paeoniae Radix Alba* samples and can visually present whether there are intuitive quality problems in *Paeoniae Radix Alba*, which provides the basis for the subsequent quality assessment and helps to quickly screen out defective samples.

In the field of grading *Paeoniae Radix Alba*, some experts use a ruler to measure the maximum diameter of the decoction pieces as the standard for grade evaluation, while others believe that the short diameter should be used as the grading standard. To facilitate the determination of *Paeoniae Radix Alba* quality grades, our research team utilized an algorithm to measure the maximum diameter, short diameter, and area of the decoction pieces. To quantitatively evaluate the performance, the error rate and accuracy are defined as Equation (13):

(13) $$\begin{array}{c} \begin{array}{ccc} \text{Error}\;\left(\%\right) & =\frac{|\text{Pred}-\text{Actual}|}{\text{Actual}}\times 100\% & ；\mathrm{Acc}\left(\%\right)=100\%-\text{Error}\left(\%\right) \end{array} \end{array}$$

As shown in Table 5, the majority of samples exhibit error rates within 5%, with only a few cases (e.g., Object 9 and Object 16) showing slightly higher errors in the short‐diameter measurement (3.7% and 16.5%, respectively). Correspondingly, the average accuracies (Long‐Acc and Short‐Acc) approach 97%, with maximum values exceeding 99% and minimum values still above 89%. The results indicate that the OpenCV‐based measurement framework performs more reliably when analyzing larger slices (≥ 16 mm), where the relative proportion of pixels is higher and the physical size represented by each pixel is smaller, thereby reducing the amplification of boundary localization errors. In contrast, small‐sized samples, such as Object 3, suffer from increased sensitivity to discretization effects, as fewer pixels represent the contour and any edge deviation is magnified in the final measurement. Nevertheless, size is not the sole determinant of accuracy. For example, Object 1 (17.0 mm) achieved a long‐diameter error of only 2.4%, whereas Object 6 (18.8 mm) reached 5.3%, suggesting that boundary clarity and contrast are more critical factors than physical scale alone. Specimens with sharp and well‐defined edges, such as Objects 5, 7, and 8, consistently exhibited errors below 2.5% and accuracies exceeding 97.5%, confirming the decisive role of edge quality in robust contour extraction.

Another important observation is that errors in long and short diameters do not always change synchronously, indicating a directional bias introduced during image acquisition or processing. For instance, Object 3 displayed a long‐diameter error of 5.6% but only 1.5% in the short diameter, while Object 10 showed the opposite trend with a long‐diameter error of 2.0% and a short‐diameter error of 7.5%. Such asymmetry can plausibly be attributed to anisotropic gradient distributions caused by uneven illumination, where strong gradients in one direction enhance edge responses while weaker gradients in the perpendicular direction lead to broken or less distinct boundaries.

This level of performance can primarily be attributed to two factors: (Chao et al. 2017) the use of a calibration board (26 mm × 38 mm) ensures stable pixel‐to‐millimeter conversion; (Liu and Ma 2020) controlled imaging conditions (background and illumination) provide high edge contrast, facilitating reliable contour extraction. Importantly, these results do not indicate overfitting. First, the measurement stage is based primarily on rule‐based geometric fitting (OpenCV pipeline) rather than a parameter‐heavy deep learning model, which minimizes the risk of memorizing training samples. Second, the dataset was split into training, validation, and testing subsets at a ratio of 7:2:1, and all reported accuracies are derived from the independent test set. Third, even under varying imaging conditions (Section 2.1.2), the system maintained stable measurement accuracy, suggesting strong generalization capability.

Compared to other studies, the precision of our method is competitive or superior. For example, Wang et al. (2018) developed the FruitSize smartphone application for orchard fruit measurement, reporting RMSE values of 2.0 to 5.5 mm, corresponding to 3% to 8% error rates for medium‐sized fruits (60–80 mm in diameter). By contrast, our system achieved ≤ 5% error on smaller herbal slices (15–30 mm), demonstrating higher relative precision due to the use of a calibrated pixel‐to‐millimeter mapping.

In terms of defect detection, the EF‐YOLO model achieves 74.1% mAP@0.5 with only 5.4 GFLOPs and 2.62 M parameters (Table 4). This performance is notably more efficient than traditional deep learning methods. For instance, Gobert et al. (2018). applied high‐resolution imaging with SVM classifiers for defect detection in metal additive manufacturing, obtaining 63% to 85% (Single mode 63%–73%, 85% after multimodal integration) accuracy depending on the sensor modality, while requiring more complex pipelines. Similarly, Zhang et al. (2024). proposed a neural network for X‐ray weld defect detection, reaching 61.8% mAP@0.5 with an absolute error of ≤ 0.1 cm. Although targeting industrial weld defects, the pixel‐to‐size mapping logic is consistent with our approach, and the results collectively highlight that our EF‐YOLO framework delivers competitive accuracy while maintaining superior computational efficiency.

In summary, the analysis in Table 5 demonstrates that for the measurement of the long diameter and short diameter of *Paeoniae Radix Alba* decoction pieces, the proposed system achieves an overall measurement error controlled within 5%; meanwhile, the accuracy rates of the vast majority of samples consistently maintain within the range of 95% to 99%. This level of performance fully satisfies the precision requirements for industrial quality inspection. At the same time, the relatively higher errors observed for smaller‐diameter or low‐contrast slices highlight potential directions for future work, such as edge enhancement, adaptive parameter tuning, and specialized optimization for small targets.

## 4 Discussion

Although our model has made significant progress in the detection of defects in *Paeoniae Radix Alba* (white peony root), there is still a long way to go before it can evolve into a generalized algorithm for the quality assessment of traditional Chinese medicinal herbs. While the current model demonstrates high accuracy and efficiency in detecting defects in white peony slices, the diversity of medicinal herbs, with their varying visual characteristics, complex shapes, and defect types, presents a significant challenge for ensuring the robustness and generalizability of the algorithm. In future work, we will focus on enhancing the model's adaptability to a wider range of medicinal herbs and ensuring that it maintains high performance in complex production environments. To achieve this, we plan to explore more advanced deep learning techniques and leverage larger, more diverse datasets to improve the model's ability to learn from complex and varied scenarios, ensuring consistent performance across different types of herbs and defects. Additionally, we will continue to optimize the model's lightweight design with the goal of deploying it as a mobile application, allowing real‐time detection on‐site in production environments. This mobile deployment will not only provide greater portability but also meet the low‐power requirements of real‐world production devices, thereby assisting traditional Chinese medicine (TCM) manufacturers in improving quality management efficiency, reducing labor costs, and advancing automation in quality control. With these improvements, we aim to develop an algorithm that not only excels in defect detection for white peony root but can also be extended to the broader field of TCM quality assessment. Ultimately, this work will contribute to the digitization and modernization of the TCM industry, enhancing quality assurance and paving the way for the global expansion of TCM products.

## 5 Conclusions

This study addresses the issue of quality detection for *Paeoniae Radix Alba* decoction pieces and proposes a lightweight and efficient detection algorithm. The algorithm employs a self‐developed lightweight detection head, LSD‐Head, combined with a feature fusion attention (FCA) mechanism, significantly reducing computational complexity while maintaining detection accuracy. Compared to the YOLOv8 model, the proposed algorithm reduces the number of parameters by 13%, to only 65.8% of YOLOv8, while improving accuracy by 2.6 percentage points. Additionally, the algorithm uses OpenCV technology to achieve high‐precision detection of the physical dimensions of *Paeoniae Radix Alba* decoction pieces, with an average measurement error controlled within 5%. Experimental results show that the proposed model not only quickly and accurately detects defects in the slices, but also effectively measures their area and maximum and minimum diameters, significantly enhancing the efficiency and precision of quality control. Compared to traditional manual detection methods, this automated detection system significantly reduces detection time and human error, providing a more efficient and cost‐effective alternative. Overall, the innovative detection model features a lightweight design, high accuracy, and low computational complexity, making it particularly suitable for resource‐constrained embedded devices, demonstrating great potential for application in the automated detection and quality management of traditional Chinese medicinal materials.

Traditional Chinese medicinal materials exert their therapeutic effects through the synergistic action of multiple compounds. Ensuring high quality is crucial for maximizing their clinical efficacy. However, traditional methods of defect detection and physical property evaluation for medicinal materials may not be comprehensive or efficient. This study explores the application of lightweight and efficient algorithms in the detection of cracks in *Paeoniae Radix Alba* decoction pieces, with a focus on accuracy and efficiency in practical applications.

## Author Contributions

**Miao Huan:** methodology (equal), validation (equal), writing – original draft (equal). **Tao Wang:** conceptualization (equal), methodology (equal), validation (equal), writing – review and editing (equal). **Qin Xu:** validation (equal). **Lu Gao:** methodology (equal). **Zhouxiang Lu:** visualization (equal). **Xingyun Shi:** investigation (equal). **Miaohua Qian:** writing – review and editing (equal). **Liangquan Jia:** conceptualization (equal), formal analysis (equal), funding acquisition (equal), project administration (equal), supervision (equal). **Chong Yao:** funding acquisition (equal), investigation (equal), resources (equal), supervision (equal).

## Ethics Statement

The authors have nothing to report.

## Consent

The authors have nothing to report.

## Conflicts of Interest

The authors declare no conflicts of interest.

## Data Availability

The datasets used and/or analyzed during the current study are available from the corresponding author on reasonable request.
